# Supplementary figures and images for: Sequences and proteins that influence mRNA processing in Trypanosoma brucei: Evolutionary conservation of SR-domain and PTB protein functions
Source: PLoS Negl Trop Dis. 2022 Oct 26;16(10):e0010876. doi: 10.1371/journal.pntd.0010876 (PMC9639853; doi:10.1371/journal.pntd.0010876)

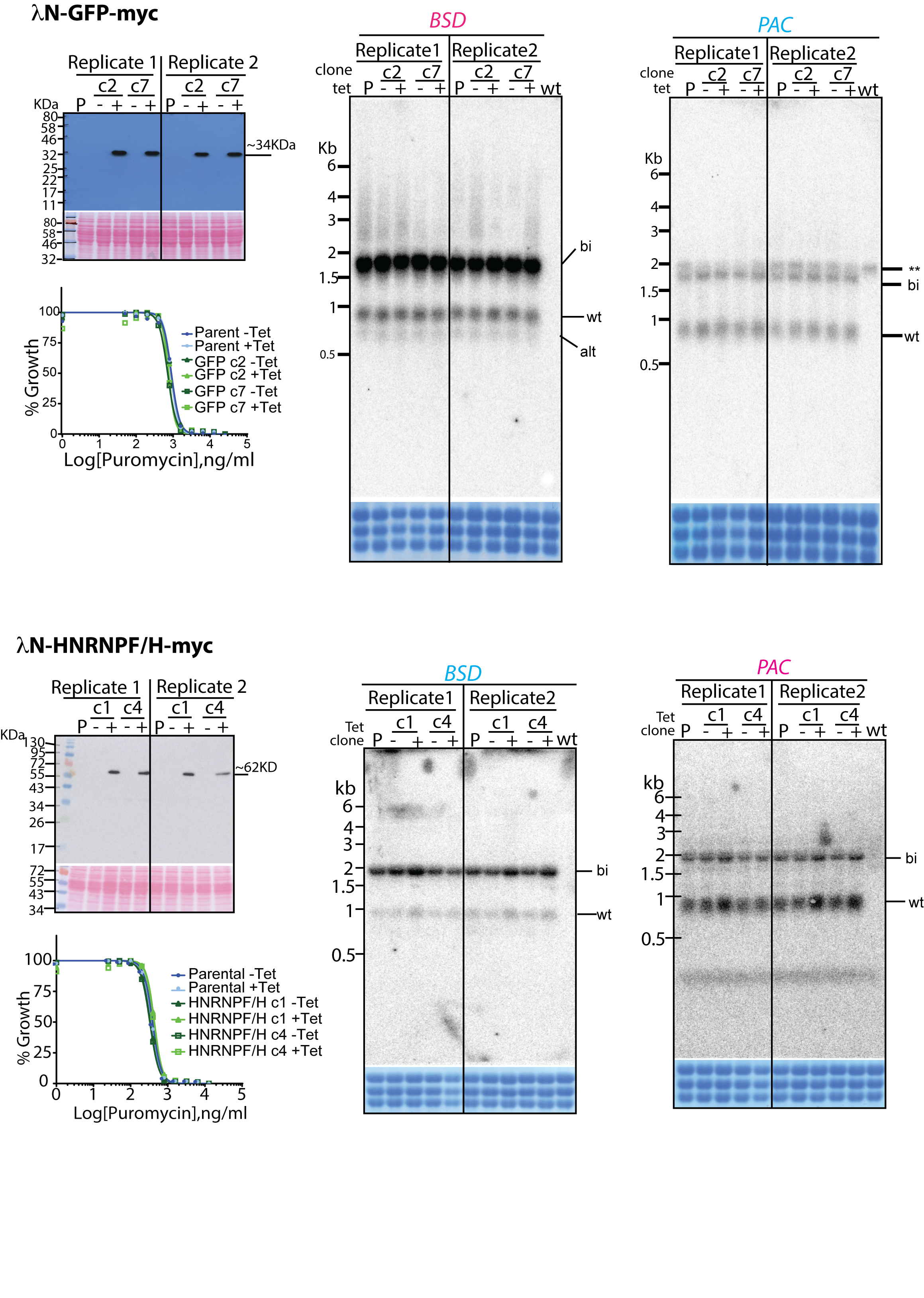

Supplement: S1 Fig — (TIF) [file pntd.0010876.s001.tif]

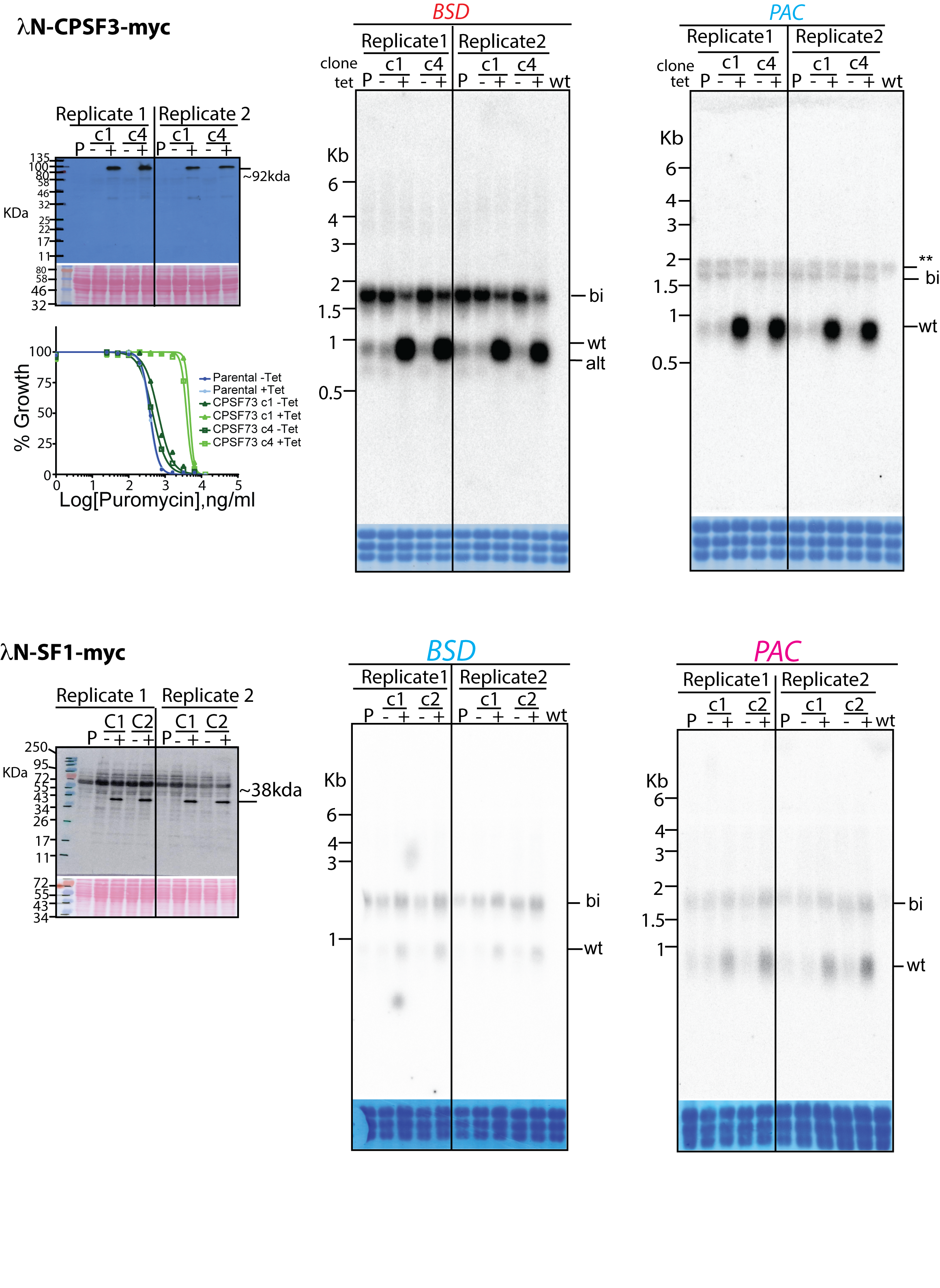

Supplement: S2 Fig — (TIF) [file pntd.0010876.s002.tif]

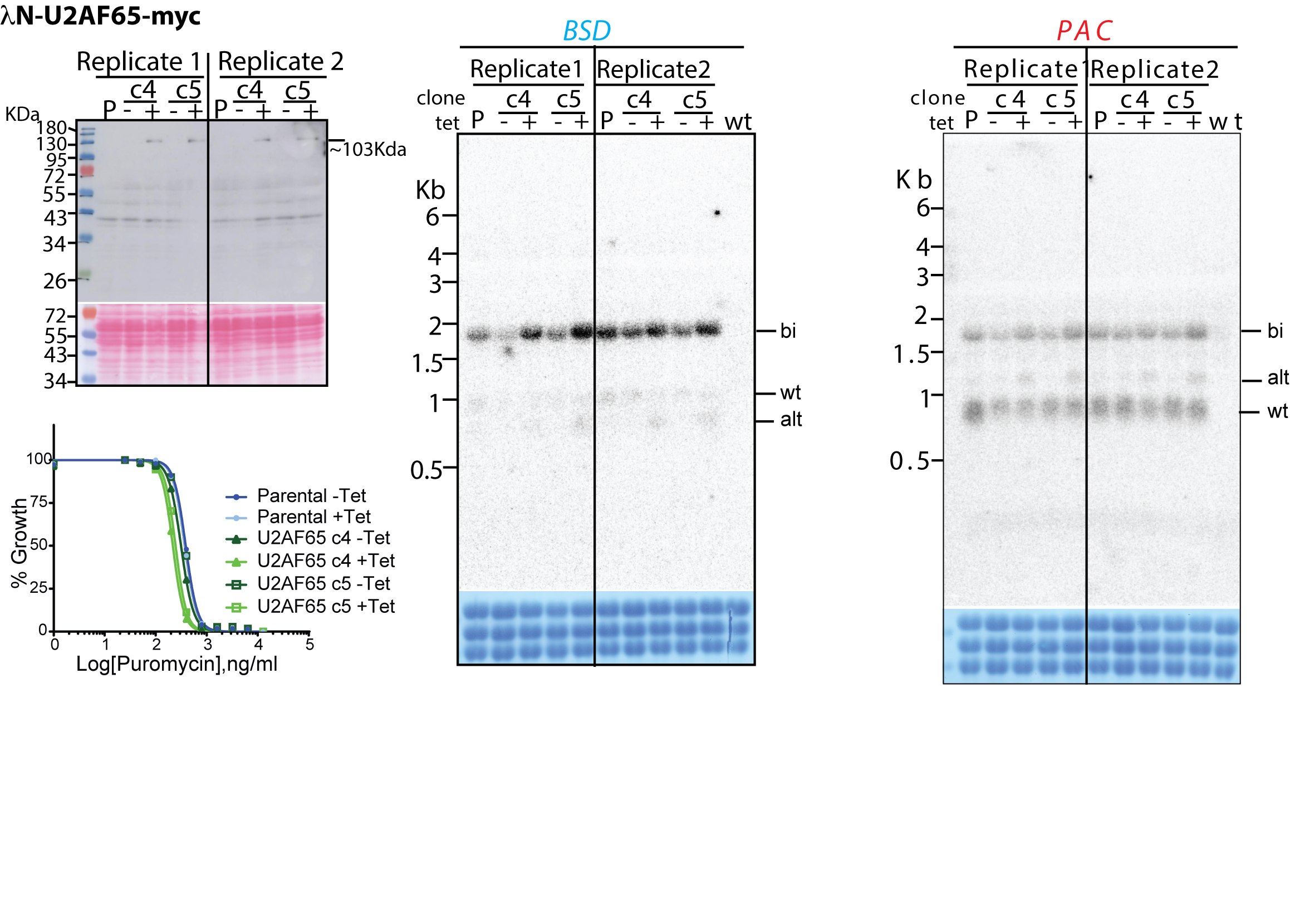

Supplement: S3 Fig — (TIF) [file pntd.0010876.s003.tif]

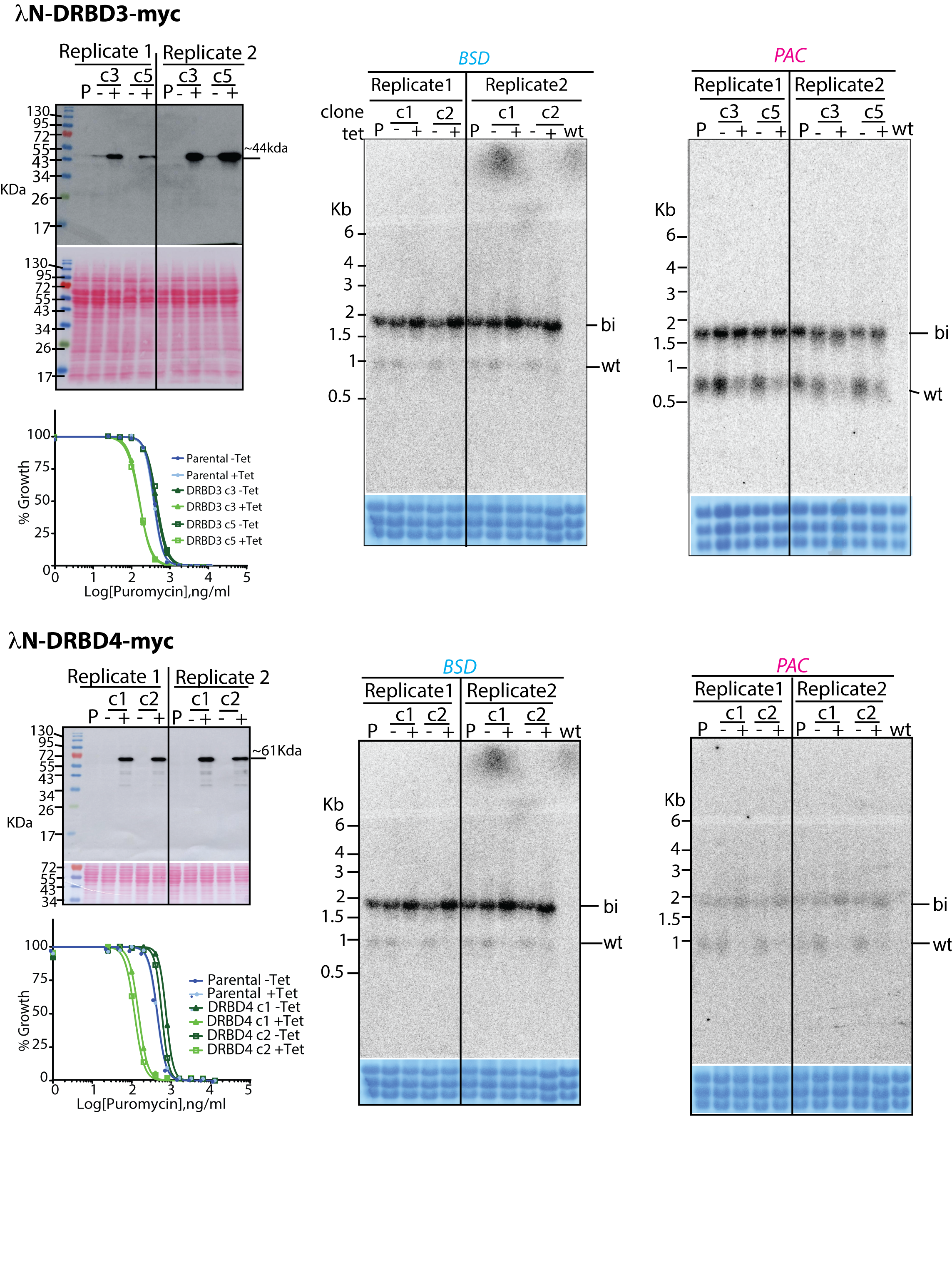

Supplement: S4 Fig — (TIF) [file pntd.0010876.s004.tif]

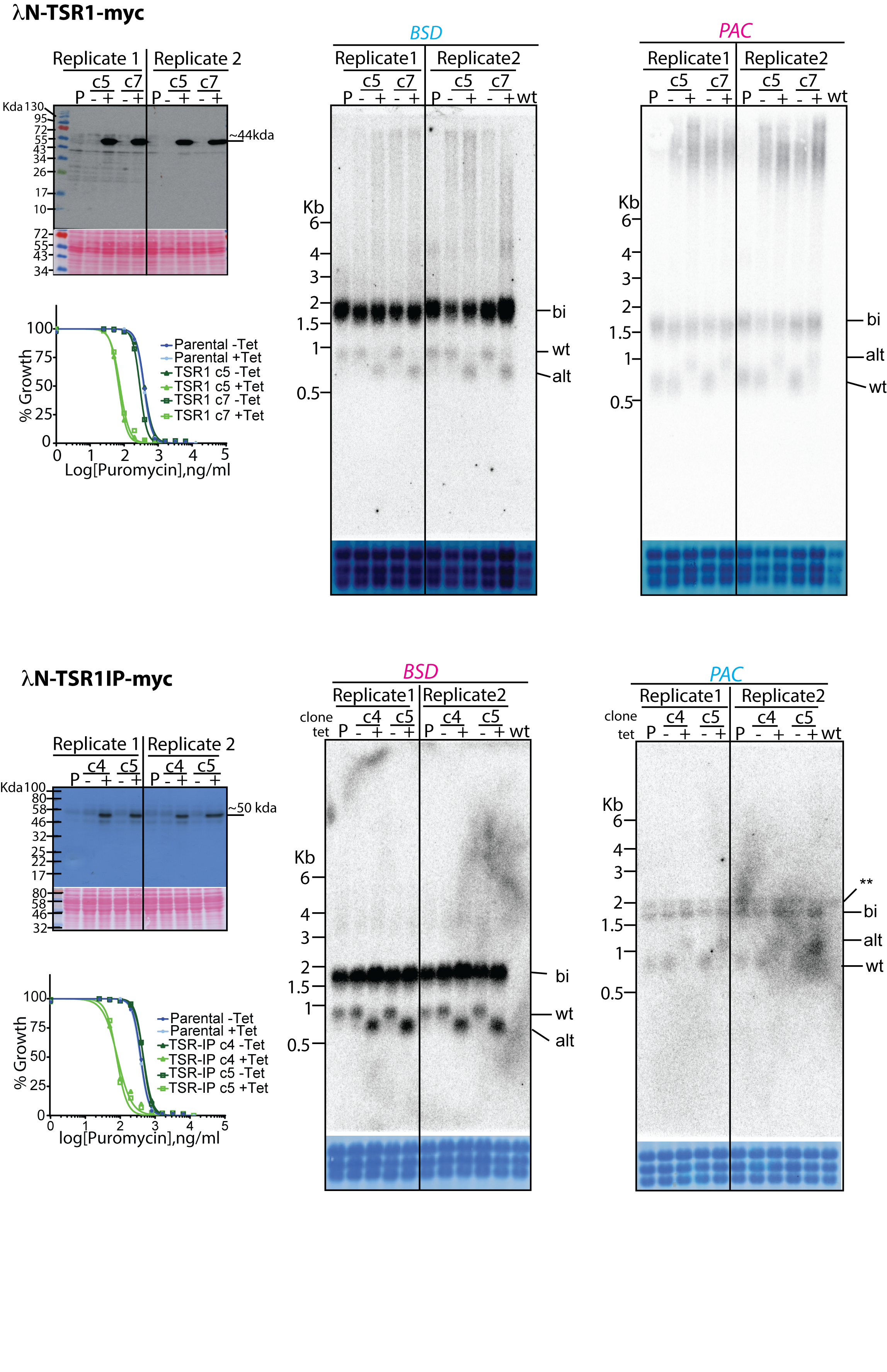

Supplement: S5 Fig — (TIF) [file pntd.0010876.s005.tif]

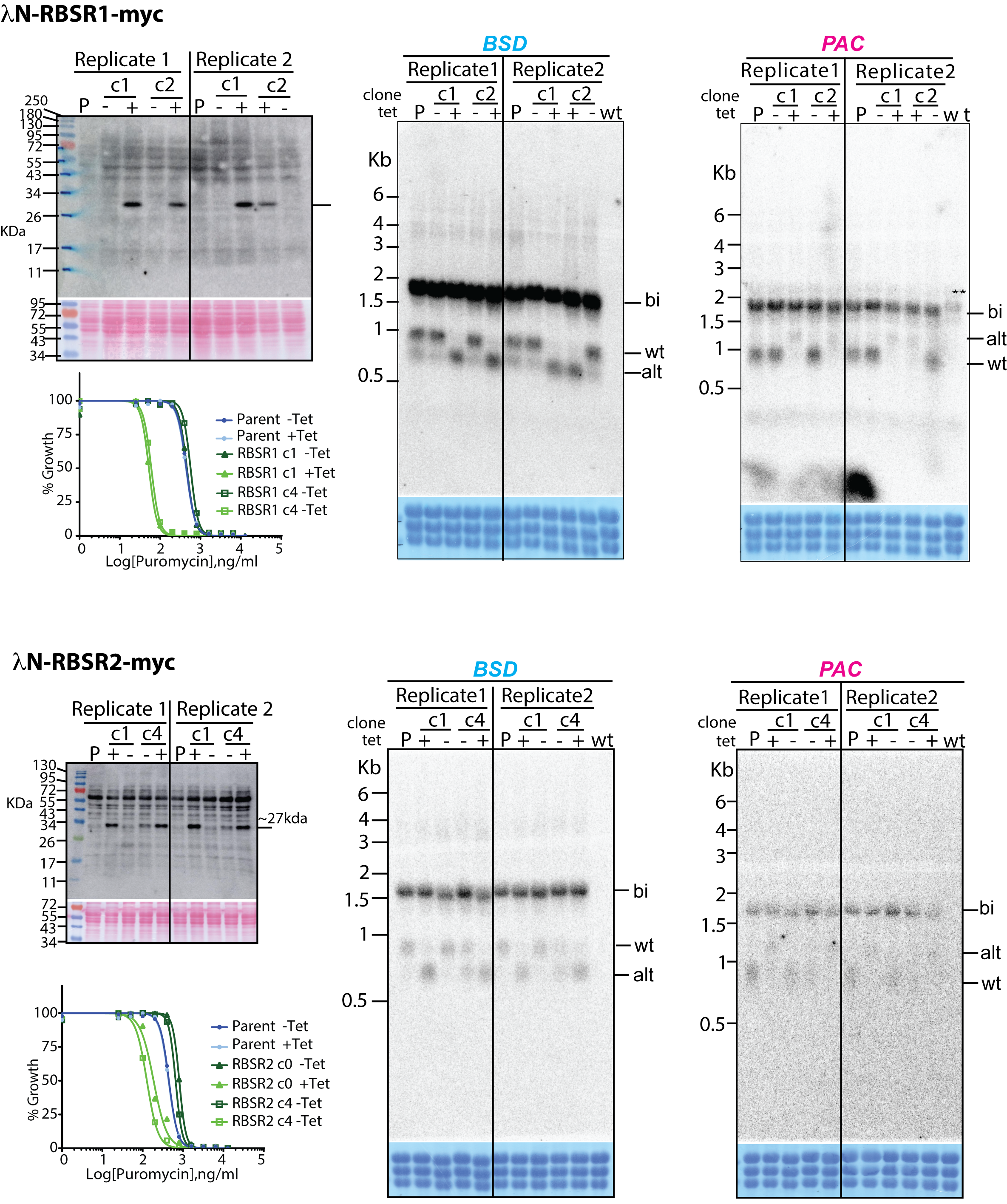

Supplement: S6 Fig — (TIF) [file pntd.0010876.s006.tif]

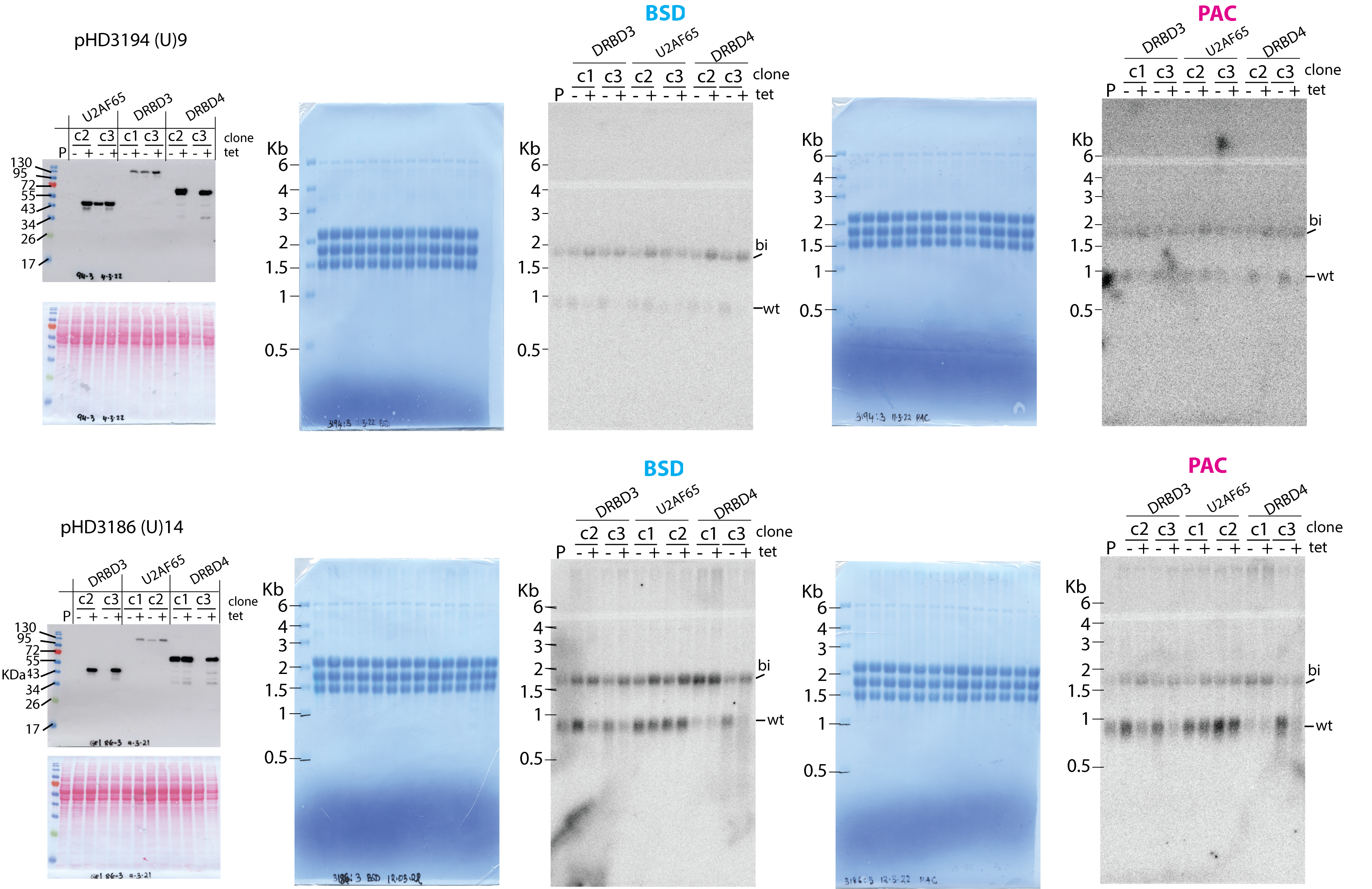

Supplement: S7 Fig — (TIF) [file pntd.0010876.s007.tif]

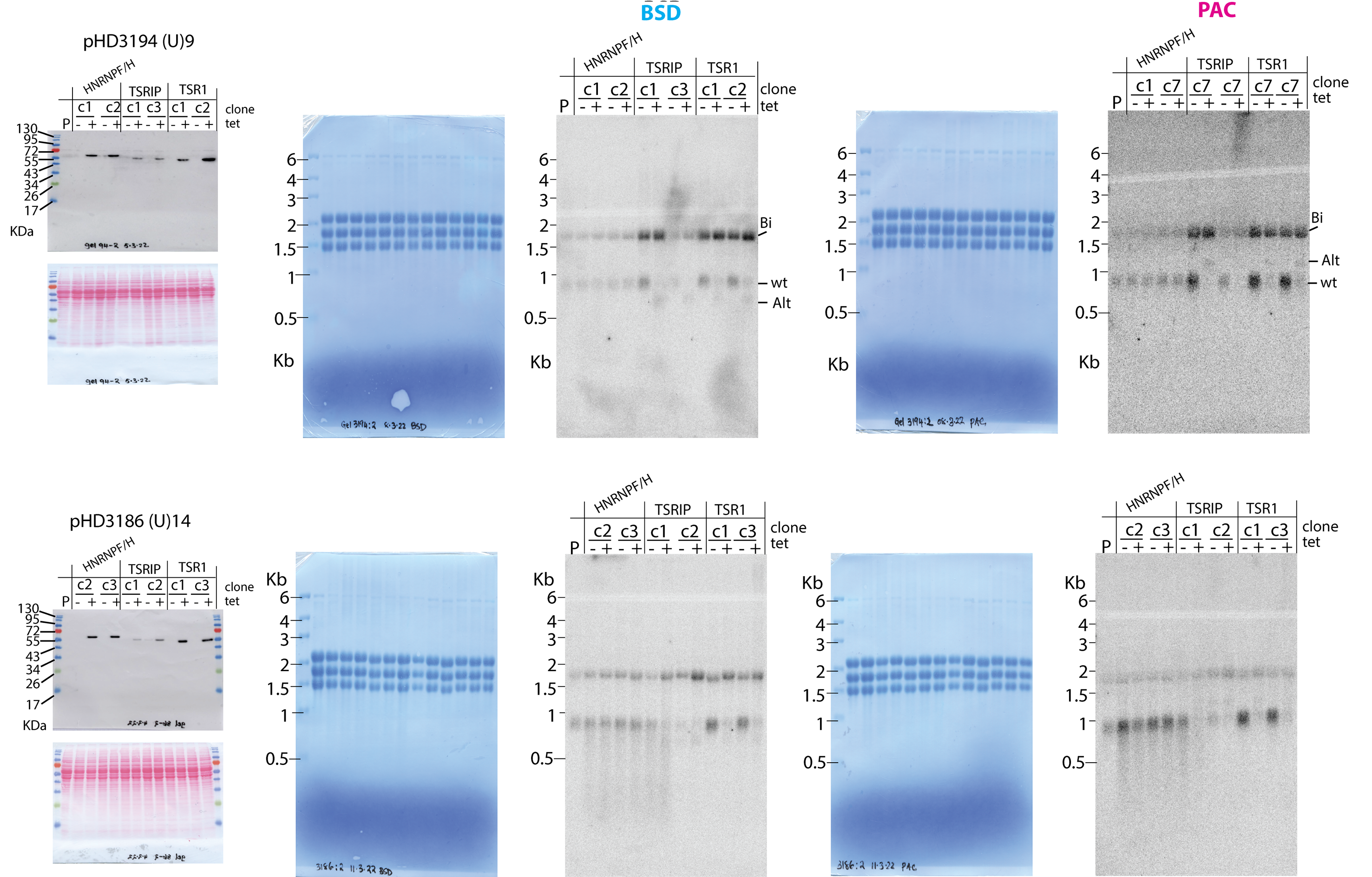

Supplement: S8 Fig — (TIF) [file pntd.0010876.s008.tif]

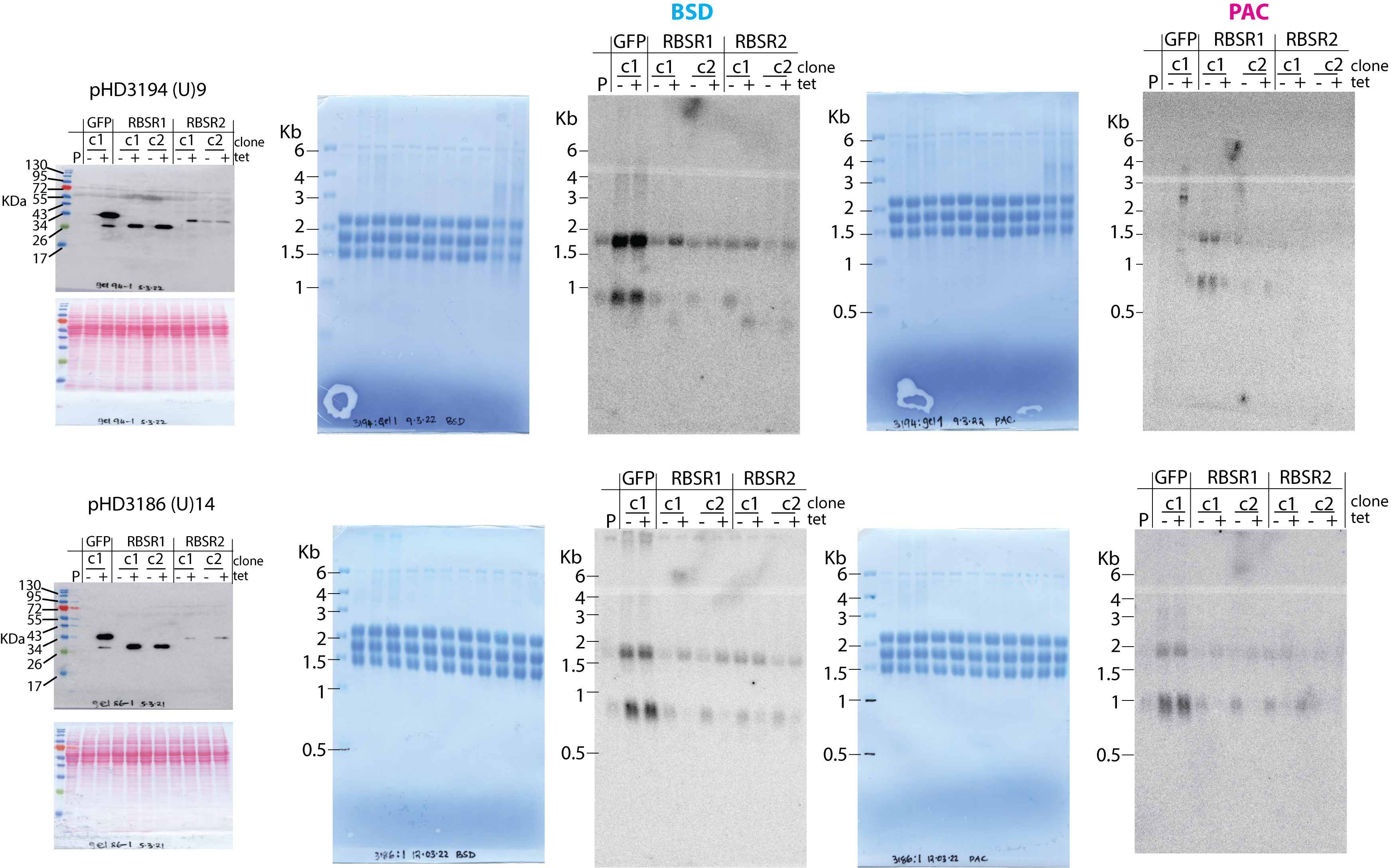

Supplement: S9 Fig — (TIF) [file pntd.0010876.s009.tif]

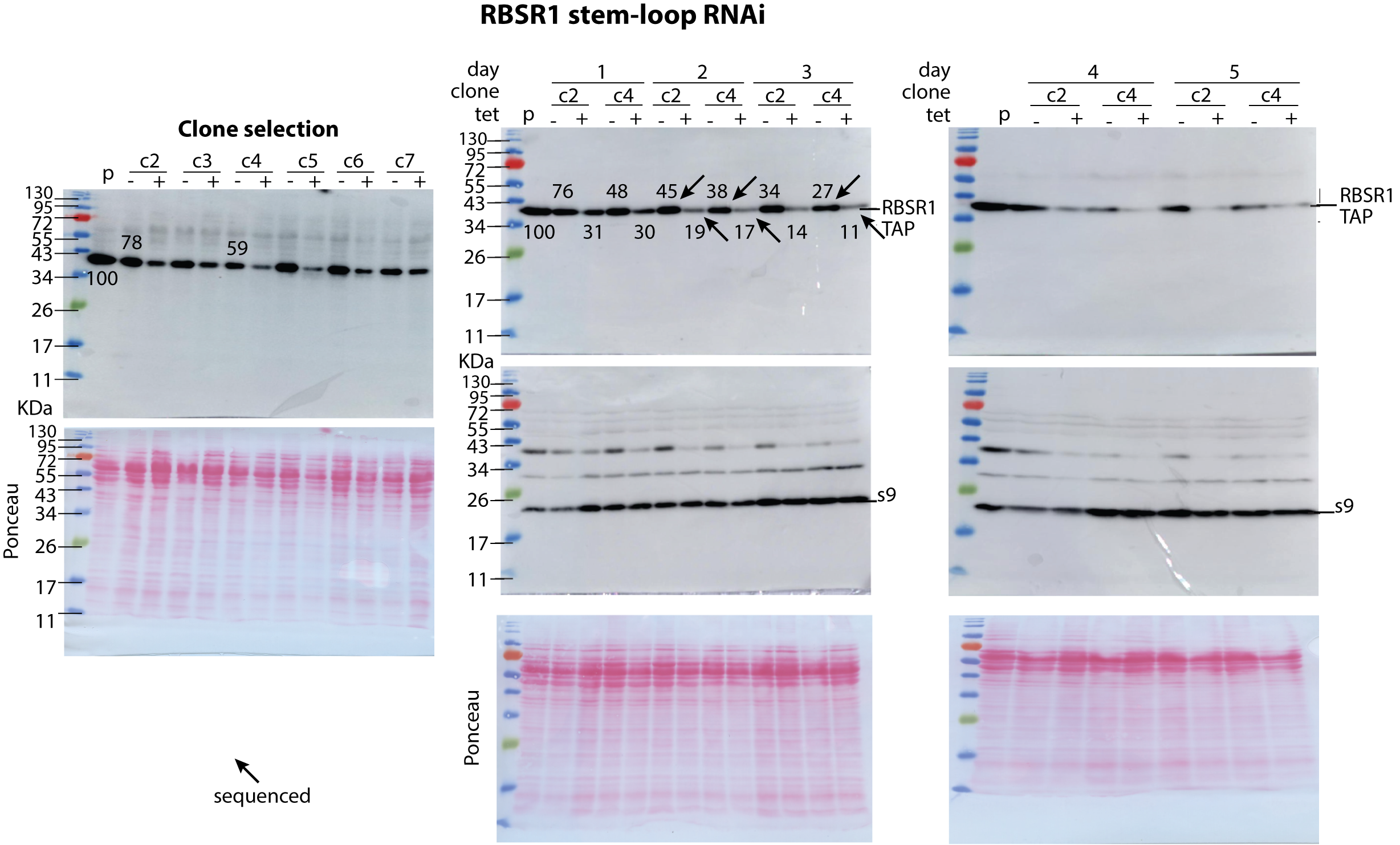

Supplement: S10 Fig — "P" is the precursor cell line with the TAP tag but no RNAi plasmid. The numbers are quantitation and the arrows indicate samples used for RNASeq. (TIF) [file pntd.0010876.s010.tif]

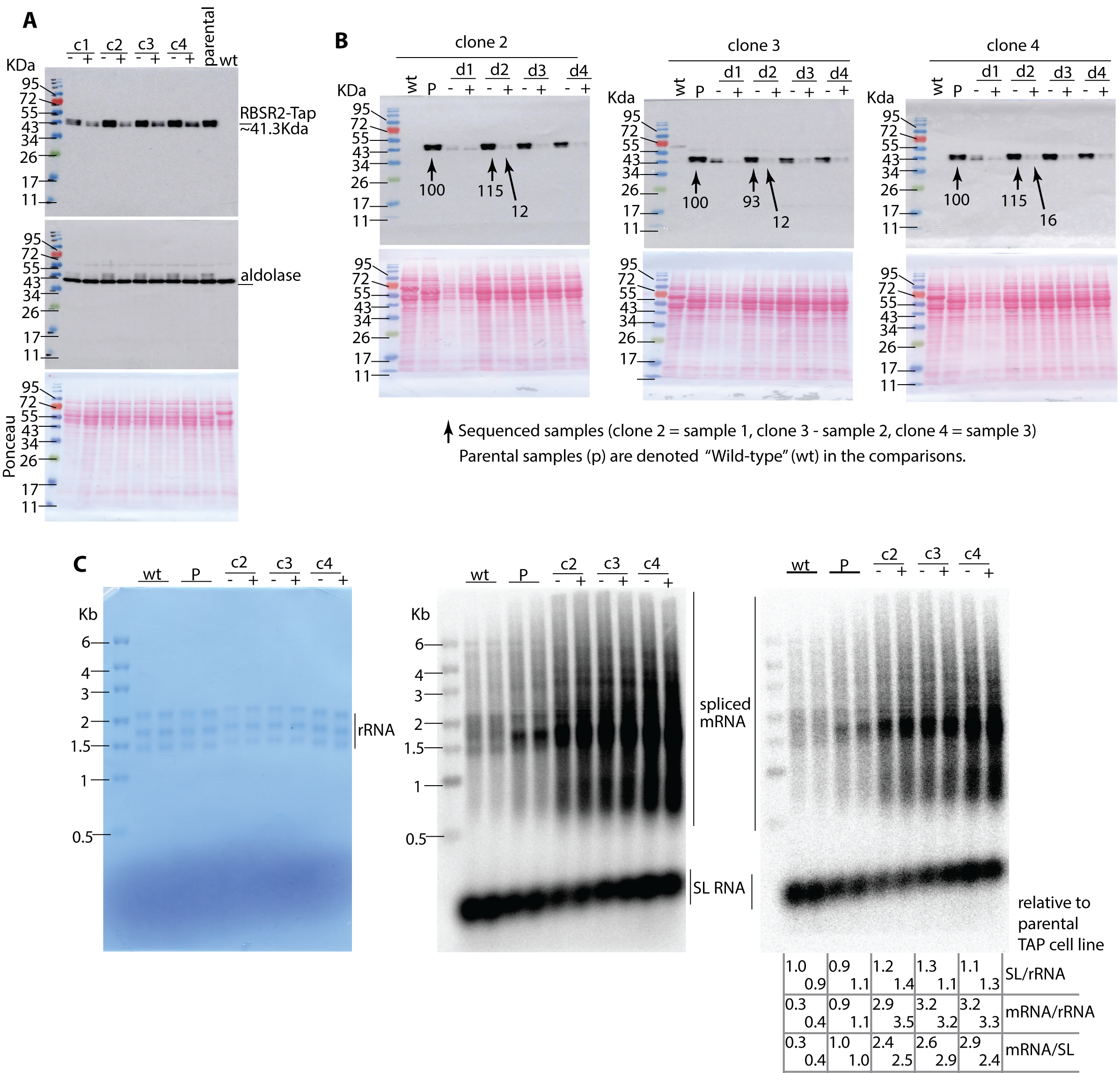

Supplement: S11 Fig — "P" is the precursor cell line with the TAP tag but no RNAi plasmid. The numbers are quantitation and the arrows indicate samples used for RNASeq. S9 is ribosomal protein S9. Panel C shows a Northern blot of the RNA used for seequencing, hybridised with a spliced leader probe. "P? is two samples of the input (tagged) line that serves as the "wild-type" control in the RNASeq analysis. "wt" is RNA from another experiment; the low amount of mRNA in these might be caused by high cell density but this is uncertain. Two exposures are shown and relative quantitation of the shorter exposure (normalised to rRNA) is shown. (TIF) [file pntd.0010876.s011.tif]

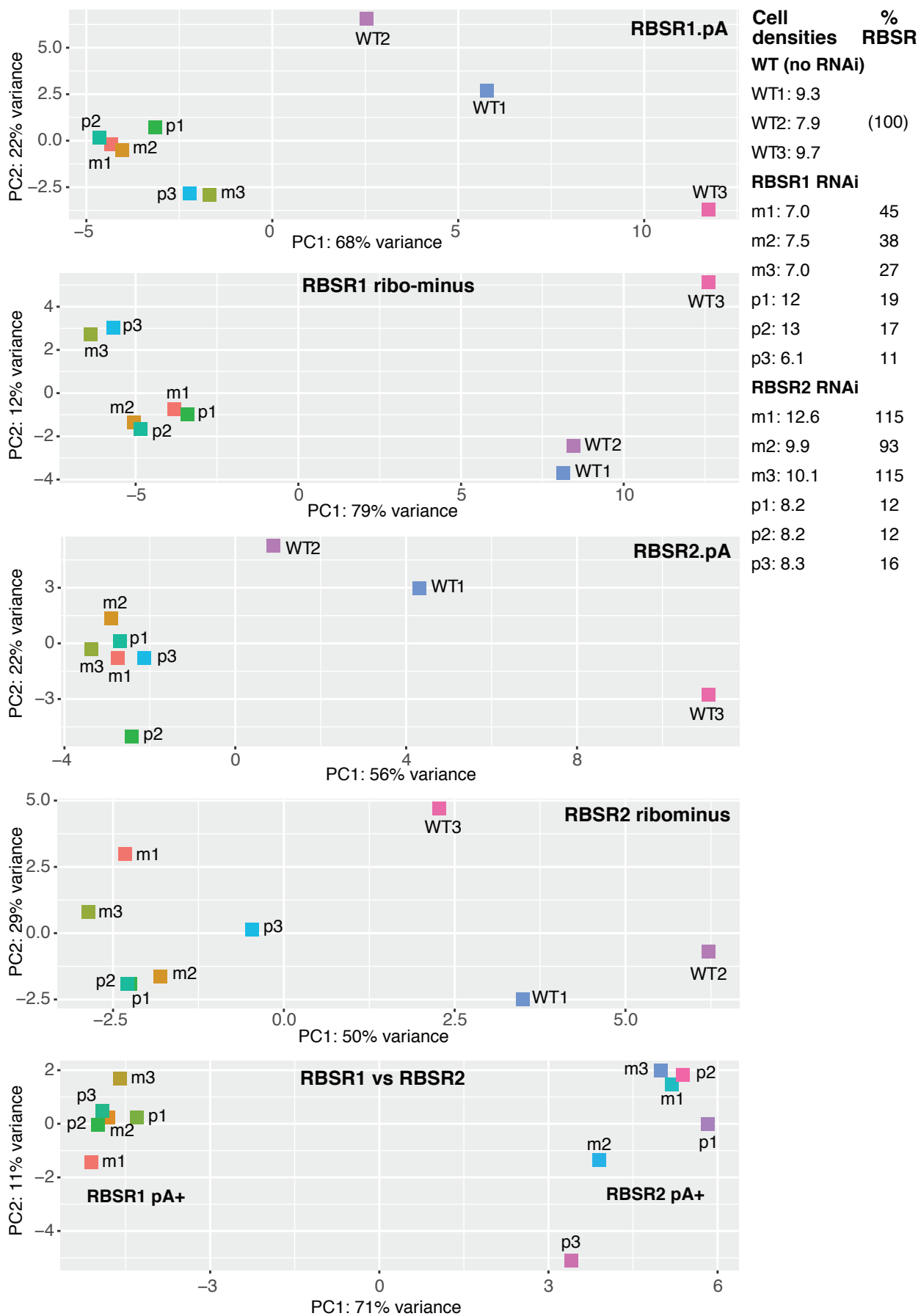

Supplement: S12 Fig — "WT" here refers to the line expressing RBSR2-TAP, without any RNAi plasmid. "m" means minus tetracycline, "p" means plus tetracycline, for the three replicates illustrated in S10 and S11 Figs. Cell densities (multiplied by 10−5) and the percent of the RBSR protein for each sample (as shown in S10 and S11 Figs) are also shown. "pA" is poly(A)+ RNA, ribominus is rRNA-depleted RNA. (PDF) [file pntd.0010876.s012.pdf]

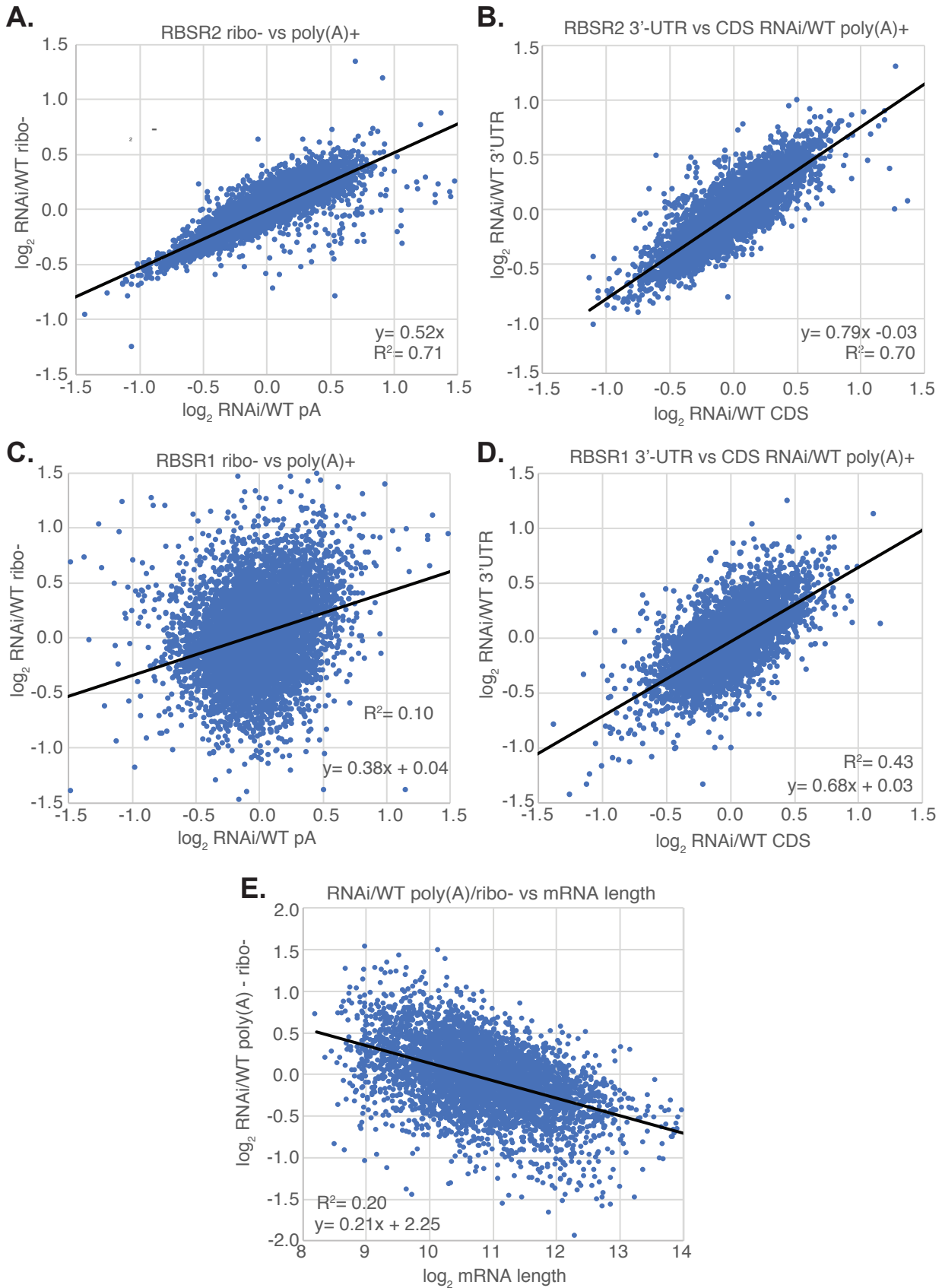

Supplement: S13 Fig — In all cases the results for +tet and -tet were pooled. All results are log2-transformed. A. RBSR2, RNAi cell line/WT, poly(A)+, on x-axis, ribo-minus on y-axis. B. RBSR2, RNAi cell line/WT, poly(A)+, coding sequence (CDS) on x-axis, 3’-UTR on y-axis. C. RBSR1, RNAi cell line/WT, poly(A)+, on x-axis, ribo-minus on y-axis. D. RBSR1, RNAi cell line/WT, poly(A)+, coding sequence (CDS) on x-axis, 3’-UTR on y-axis. E. RBSR1, RNAi cell line/WT, poly(A)+ result divided by ribo-minus result R on y-axis, mRNA length on x-axis. (PDF) [file pntd.0010876.s013.pdf]
