# Supplementary material for: Sequences and proteins that influence mRNA processing in Trypanosoma brucei: Evolutionary conservation of SR-domain and PTB protein functions
Source: PLoS Negl Trop Dis. 2022 Oct 26;16(10):e0010876. doi: 10.1371/journal.pntd.0010876 (PMC9639853; doi:10.1371/journal.pntd.0010876)
Supplement: Attachment — Submitted filename: PNTD replies.docx [file pntd.0010876.s026.docx]

Reviewer's Responses to Questions

Reviewer #1: The objectives of the study clearly articulated in lines 109 -110 and the study design appropriately addresses the stated objectives. However, it adds clarity to provide a citation or document how the radiolabelled probes were generated (line 146).
>>added

Reviewer #3:
Since the model used (transgenic L427) is a trypanosome clone which has been maintained in culture for many years and which, therefore, probably can no longer grow in the mammalian host, the authors should be cautious in extrapolating observations made here to the trypanosomes growing in the mammalian host.

>>A note to this effect has been added

Alibu et al. does not appear in the list of references  (line 116).
>>Fixed
--------------------

Reviewer #1: The entire reporter construction section spanning lines 182 to 198 is more of a method and less of a result and should be placed correctly within the methods section especially considering that the PPT library preparation section depends on the constructed pHD3180 plasmid.

>>We have added a description in the Methods section but retained most of the text in the results section since it is critical to understanding of the experiments and some people will not read the Methods.

Reviewer #3 The authors should explain, in the appropriate section of the manuscript, the differences in average size of the RNA in Fig. 3, lanes 1and 2, ACT, BSD. They seem to be identical in size under PAC (line 284).

>>Many more details have been added.

Furthermore, there should be an explanation of the bases upon which the predictions were made regarding the faster than average splicing, and slower processing of some transcripts referred to here (lines 294-295).

>>Many more details have been added.

What is the statistic supporting the significance of level of homology? Either cite a reference or provide an explanation

>>we assume this refers to the possible identification of DRBD3/4 with PTBs. It's rather complicated, and based both arrangement of the RRMs, and their sequences. We have added an extra sentence to this effect.

--------------------

Reviewer #3: The discussion is too long and rambles on without clearly bringing out the key contribution of the work. It would be most helpful to state concisely and discus the key features of this potentially useful vector, and its general applicability in studying trans-splicing in trypanosomes, etc. In particular, there is only scanty reference to figures which may be relevant to the point(s) being discussed. This leaves it to the reader to guess which figure the authors have in mind when making a particular point in the Discussion.

>>We have tried to improve the clarity, and shortened some sections. **NOT FINISHED**
--------------------

Reviewer #1: The various Trypanosoma brucei homologues of splicing factors or regulators (CPSF3, SF1, DRBD3, DRBD4, RBSR1, RBSR2,TSR1,HNRNPF/H, U2AF65) should be clearly named in Lines 109 -110 such that a reader is not left guessing.
>>Done

Reviewer #1: The section that begins"Two Life cycles stages of T. brucei grow well in vitro: the mammalian "bloodstream" form, and the "procyclic" form which grows in the midgut of tsetse flies  is misplaced and should be positioned around or just before line 70.
>>Done

Delete "that" appearing at the beginning of line 261.
>>Done

Reviewer #2:
The system has its weaknesses, in particular for larger screens, and the authors are aware of it. Tethering data are always a bit tricky as the orientation matters. The output (antibiotica resistance) is not only determined by splicing efficiency, but can also be affected by differences in export and translation efficiency and it is not well known how such dicistronic transcripts behave in trypanosomes in this regard. This means, that the system still requires northern blots for data interpretation, which are not high throughput. For these reasons, this reviewer is not convinced that the system is very suitable for further screens.

>>We agree that Northern blots would be required for detailed analysis and for confirmation of screen results. But that doesn't invalidate the potential of the system for initial screening. All high-throughput screens have weaknesses and require individua verification. If you change the 5'-UTR you change the translation - which is also, then, a valid read-out. We now moved the description of the tehering assay to the introduction, in order to state both the advantages and disadvantages up-front.

However, the data are still highly interesting, as they contain a few conclusions about splice sites in trypanosomes that are new. They support the current model that trypanosomes often “find a way” to splice their mRNAs even with sub-optimal splice sites. And they provide a tool to produce dicistronic mRNAs, perhaps for further studies of the
subcellular localization and mRNA export control in trypanosomes.

I do have a few comments:

Figure 1 C-E
It would be easier for the reader, if the authors could include the data from E to C and D, rather than showing a wrong clone (its already rather complex). The difficulties to get the clone can be discussed in the text (as they are meaningful) and perhaps the data can also be shown in sup. Material.

>>We looked into this since it would indeed be easier to describe. Unfortunately, the signal from the "wrong" clone is really intense and spills over into the neighbouring lanes, so it's impossible to remove it cleanly. Cutting bits out of the gel to selectively remove the shadow would have been unacceptable.

Figure 1, C and D
I would expect that the splicing efficiency with the same PTT is equal, independent on whether the Northern blot is probed for BSD or PAC. But it looks like PAC monomer per BSD-PAC dimer is a lot more than BSD monomer per BSD-PAC dimer. Can the authors provide an explanation? Is the BSD mRNA much less stable than the PAC mRNA? And if so, how does it impact the screen?

Good point, it's probably the half-lives, actin mRNA is 3 times less stable than the tubulin mRNA. The text has been modified to include this information.

The authors show that the dicistronic RNA gives poor blasticidin resistance, possibly because the PAC sequence in the ‘UTR’ interferes with either translation or nuclear export. Still, they use blasticidin selection in their cis-element PPT screen to obtain the clones, that are then, in a second step, challenged with puroymcin selection to look for splicing supportive or suppressive sequences. How can the authors be sure that splicing-suppressive sequences are not lost in the blastidicin selection step already?

We can't, note added in the text.

RBSR1 / RBSR2 dataset: It doesn’t really fit into the story... Consider to publish as individual manuscript? Or, if not possible, restructure a little bit, last part of the manuscript application to the unknown, 1) interaction data and 2) test for splicing efficiency.

We would definiteily have done this if we had seen clear effects after the RNAi or KO. However, the effects were basically uninterpretable, which would have made separate publication extremely difficult. We prefer to keep the order as it is since it actually reflects the true logic of the experiments: we did the tethering first, and followed up because we got an interesting result. some alterations have been made to reflect this. (We actually also tried to tether RBSR3as well, for completeness, but didn't manage to get the cell line.)

Data Requirements:
Please note that, as a condition of publication, PLOS' data policy requires that you make available all data used to draw the conclusions outlined in your manuscript. Data must be deposited in an appropriate repository, included within the body of the manuscript, or uploaded as supporting information. This includes all numerical values that were used to generate graphs, histograms etc.. For an example see here: <http://www.plosbiology.org/article/info%3Adoi%2F10.1371%2Fjournal.pbio.1001908#s5>.

All gels images are already complete. The excel tables are on Figshare and the mass-spec and trasncriptome data are also available.
