# Supplementary material for: Sequences and proteins that influence mRNA processing in Trypanosoma brucei: Evolutionary conservation of SR-domain and PTB protein functions
Source: PLoS Negl Trop Dis. 2022 Oct 26;16(10):e0010876. doi: 10.1371/journal.pntd.0010876 (PMC9639853; doi:10.1371/journal.pntd.0010876)
Supplement: Attachment — Submitted filename: Reviewer Comments on PNTD-D-22-00915.pdf [file pntd.0010876.s025.pdf]

Reviewer’s comments on manuscript PNTD-D-22-00915, entitled:

Sequences and proteins that influence mRNA processing in *Trypanosoma brucei*: evolutionary conservation of SR-domain and PTB protein functions

General

The authors report on investigations on factors which influence the choice of site for trans-splicing of a capped leader sequence at the 5’end of mRNA in *Trypanosoma brucei.* For the investigations, the authors used a transgenic clone of *T. brucei* maintained by growth in culture.

The paper is in part a methods one, in which the authors describe approaches to finding polypyrimmidine tracts (PPTs) active in the trans-splicing in trypanosomes. Since the model used (transgenic L427) is a trypanosome clone which has been maintained in culture for many years and which, therefore, probably can no longer grow in the mammalian host, the authors should be cautious in extrapolating observations made here to the trypanosomes growing in the mammalian host.

The manuscript is well-written and is sufficiently detailed, only requiring a few improvements, as suggested below:

Specific

Section	Line #	comments
**Abstract**		
**Introduction**	56	Move reference to Figure 1 to the Results section
	95-98	Break this sentence into two parts for clarity
**Methods**	116	Alibu et al. does not appear in the list of references.
**Results**	284	The author should explain, in the appropriate section of the manuscript, the differences in average size of the RNA in Fig. 3, lanes 1and 2, ACT, BSD. They seem to be identical in size under PAC.
	294-295	The authors should explain the bases upon which the predictions were made regarding the *faster than average splicing,* and *slower processing* of some transcripts referred to here.
	370	What is the statistic supporting the significance of level of homology? Either cite a reference or provide an explanation
**Discussion**		The discussion is too long and rambles on without clearly bringing out the key contribution of the work. It would be most helpful to state concisely and discus the key features of this potentially useful vector, and its general applicability in studying trans-splicing in trypanosomes, etc. In particular, there is only scanty reference to figures which may be relevant to the point(s) being discussed. This leaves it to the reader to guess which figure the authors have in mind when making a particular point in the Discussion.

Specific suggestions/comments are below.

Conclusion

Accept after revision
